# Accessible mathematics videos for non-disabled students in primary education

**DOI:** 10.1371/journal.pone.0208117

**Published:** 2018-11-28

**Authors:** Alejandro Rodriguez-Ascaso, Emilio Letón, Jaime Muñoz-Carenas, Cecile Finat

**Affiliations:** 1 aDeNu research group, Universidad Nacional de Educación a Distancia (UNED), Madrid, Spain; 2 Department of Artificial Intelligence, Universidad Nacional de Educación a Distancia (UNED), Madrid, Spain; 3 Department of Science and Technology, Centre of Educational Resources, Organización Nacional de Ciegos Españoles (ONCE), Madrid, Spain; Fondazione Ugo Bordoni, ITALY

## Abstract

Our work applies Universal Design criteria for producing and using Mathematics videos for primary education students, at a time when many countries are shifting towards inclusive education policies. We have focused on how the accessibility criteria used for students with visual impairments might affect non-disabled students. For this, we reviewed applicable Universal Design principles as well as best practices in multimedia learning. We took into account the roles, procedures, tools and standards involved in the multimedia lifecycle. We then undertook an experiment consisting of producing two videos about prime numbers with the same pedagogical contents; one video was accessible for students with visual impairments and the other one was not accessible to them. We conducted a trial in real world school settings with 228 non-disabled children, who were randomly assigned a version, either accessible or not accessible, and were then asked to take a test to measure objective aspects of their learning concerning retention and transfer as well as several subjective aspects, including the attractiveness of the videos. Results indicate that there were no significant differences in the scores obtained by students using either video, although the group who watched the accessible video obtained higher score medians in the retention questions. Moreover, students found the accessible video significantly more attractive (p = 0.042). Our study provides recommendations for different stakeholders and stages within the process of producing multimedia mathematics materials that are accessible to primary students with visual impairments, as well as evidence demonstrating that everybody can benefit from the recommendations for developing good quality, accessible multimedia material.

## Introduction

Multimedia material is being used more and more as a learning resource [1–4]. It is known that this kind of material, if designed effectively, can enhance learning ([5,6]; [7][8]). Furthermore, the use of teacher-created videos may increase significantly in the near future. Relevant educational paradigms include “the flipped classroom” [9] where “with teacher-created videos and interactive lessons, instruction that used to occur in class is now accessed at home, in advance of class, that becomes the place to work through problems, advance concepts, and engage in collaborative learning”, and MOOCs (massive open online courses) [10], which are “based on multimedia collections”. More specifically, the use of multimedia for teaching mathematics in primary education has been previously investigated in [11] and in [12].

The use of multimedia material should also benefit students with disabilities. According to the Salamanca Statement of UNESCO, “those with special educational needs must have access to regular schools which should accommodate them within a child-centred pedagogy capable of meeting these needs” [13]. In agreement with this statement, the education policies of many countries are shifting towards inclusive education [14]; [15]). As an example of the impact this trend is causing in the accessibility of multimedia materials, by early 2015 advocates for the deaf filed federal lawsuits against Harvard and M.I.T., citing violations of the Americans with Disabilities Act (ADA) by failing to provide closed captioning for the multimedia material in their MOOCs. This led to a settlement of the Justice Department with edX Inc. to comply with ADA and WCAG 2.0 [16] within 18 months [17]. The current situation is that edX courses have captions, the latest version of the edX multimedia player includes the option to enable/disable captions [18], and edX authors are provided with guidelines on how to create accessible multimedia [19].

The theory of Universal Design for Learning (UDL) [20] and its subsequent guidelines (UDL Guidelines 2.0 [21]) provide guidance to identify and remove barriers for all students from teaching methods and curriculum material. UDL applies in the learning context the principles of Universal Design (UD) [22], i.e., equitable use, flexibility in use, simple and intuitive to use, perceptible information, tolerance for error, low physical effort, and size and space for approach and use. In particular, adopting a User Centered Design (UCD) [23] approach for the production of all learning materials, whether they are electronic or tangible, and providing adaptations for learning materials where necessary, constitute key strategies to make elearning accessible to all, and involve students, teachers and most of the stakeholders within the elearning arena [20,24–26][27] [28] [29]. However, “simply encouraging, or even mandating, the implementation of inclusive education practices does not guarantee improved outcomes”, while training and supporting school managers and teachers adequately constitute key levers for making real inclusion to happen [30].

Furthermore, previous studies indicate that inclusive education shows neutral to positive effects on students both with and without disabilities, e.g. generic studies such as [31,32], as well as studies on learning through multimedia, such as [33].

Within the described scope, the general objective of our work is to assess the effect that accessible multimedia materials on Mathematics have on the learning process of non-disabled students. For doing so, we have carried out an experience consisting of: a) producing two multimedia objects with exactly the same pedagogic content, one of them produced without accessibility in mind (video B), while the other one is accessible for students with visual impairments (video A); b) assessing the quality of the learning achieved with each of the two videos A and B through an anonymous evaluation conducted in real settings with 228 non-disabled students in three primary schools.

To achieve the objective mentioned above, the present paper has been structured as follows: we first review applicable Universal Design principles as well as best practices in multimedia learning, both from the student and from the professional viewpoints, and define the purpose of the study (section 2). Next, we describe the multimedia materials and the tools used in our experience (section 3). Then, we describe the methodology of our experience (section 4). Next, we discuss the analysis and results of the experience and provide recommendations for different stakeholders (section 5). Finally, we come to the conclusions derived and future work (section 6).

## Accessible multimedia materials

### Related work

In this section we review the corresponding literature, techniques and standards applying to each of the principles of Universal Design that are applicable to our study, namely: perceptible information, equitable use and low physical effort.

The principle of **perceptible information** establishes that “the design communicates necessary information effectively to the user, regardless of ambient conditions or the user's sensory abilities”, see [22]. There exist international *de facto* standards on the accessibility of electronic content, such as WCAG 2.0 [16]. The standard includes the following applicable guidelines:

- Use of Color: Guideline 1.4.1 reads “Color is not used as the only visual means of conveying information, indicating an action, prompting a response, or distinguishing a visual element”.
- Contrast: Guideline 1.4.3 reads “The visual presentation of text and images of text has a contrast ratio of at least 4.5:1”. Guideline 1.4.6 is identical to Guideline 1.4.3, except for that the required contrast is higher (7:1).
- Visual presentation. Guideline 1.4.8, as for displaying multimedia, establishes that “for the visual presentation of blocks of text, a mechanism is available to achieve the following: a) Width is no more than 80 characters or glyphs; b) Text is not justified (aligned to both the left and the right margins); c) Line spacing (leading) is at least a space-and-a-half within paragraphs, and paragraph spacing is at least 1.5 times larger than the line spacing”.
- Perception of audio. Guidelines 1.4.2 (audio control) and 1.4.7 (low or no background audio). In a context like ours where the sound coming out from the loudspeakers of the classroom is adjusted by the teacher and not by the end-users (students), these guidelines recommend “to ensure that the background sounds are at least 20 decibels lower than the foreground speech content”.
- Audio description or media alternative. Guideline 1.2.3 establishes that “an alternative for time-based media or audio description of the prerecorded video content is provided for synchronized media, except when the media is a media alternative for text and is clearly labeled as such”. Audio description (AD) is the most widespread practice to describe videos orally, and can be defined as the “verbal depiction of key visual elements in media and live productions, where the description of media involves the interspersion of these depictions with the multimedia’s original audio” [34]. The benefits of AD for people with visual impairments have been reported elsewhere in the literature [35]; [36], as well as its benefits for their learning activities [37,38].

The principle of **equitable use** establishes that [22] the design should be “useful and marketable to people with diverse abilities”. Our study focuses on assessing the effect that a particular design has on non-disabled students. In the case of AD, its beneficial effect on people without visual impairments has been addressed elsewhere [39]; [33]). Furthermore, the Equitable use principle also states “regardless of ability, all users should find the design appealing to use” [22]. In line with this, principle III of UDL Guidelines 2.0 includes the following generic statement: “affect represents a crucial element to learning, and that learners differ markedly in the ways in which they can be engaged or motivated to learn”. Like in [40] we believe that the visual aspect of the design has to do with this part of the principle. In this respect, [41] established that the visual design of multimedia material has an affective function, in addition to the cognitive aspects addressed by Mayer and colleagues in the Cognitive Theory of Multimedia Learning (CTML). Furthermore, [42] showed that the visual design of multimedia learning environments ¨was able to induce positive emotions that in turn facilitated comprehension and improved performance”. In line with this, the children's attitude to the use of technology in education has been studied in the literature [43].

The principle of **low physical effort** establishes that “the design can be used efficiently and comfortably and with a minimum of fatigue” ([22]). Similarly to the approach taken by [40], in our multimedia context we are considering “the cognitive effort required to use the design efficiently, comfortably and with a minimum of fatigue”, in line with applicable aspects of the (CTML) proposed by Mayer and colleagues, and more specifically with the temporal-contiguity effect described in [44] and referenced in the multimedia elearning classification produced in [45]. This effect refers to multimedia learning enhancement when visual and spoken materials are temporally synchronized, that is, presented simultaneously rather than successively. Authors of [44] measure two different learning indicators: retention, which is the ability to remember material at some later time in much the same way it was presented during instruction; and transfer, which is the ability to use what was learned to solve new problems, answer new questions or facilitate learning new subject matter [46].

Also in relation with the principle of low physical effort, videos with aural description of the visual information have a longer duration than those without it, and this could have an effect on learning, as educational experts agree that video is best shown in short segments so as to maximize concentration [47].

### Purpose of the study

The objective of our work is to assess the effect that accessible multimedia materials on Mathematics have on the learning process of non-disabled students in primary education. In agreement with the related work described above, both objective (retention and transfer) and subjective indicators have been measured.

### Materials

This section presents the pedagogic and accessibility aspects of the multimedia materials that have been used in our experience. To carry out the research, two versions of a video (A, accessible, B, not accessible) about prime and composite (non-prime) numbers were developed:

- Video B: http://youtu.be/52O1T7EFVBc
- Video A: http://youtu.be/lCsWVByY7Gk

**Fig 1:**
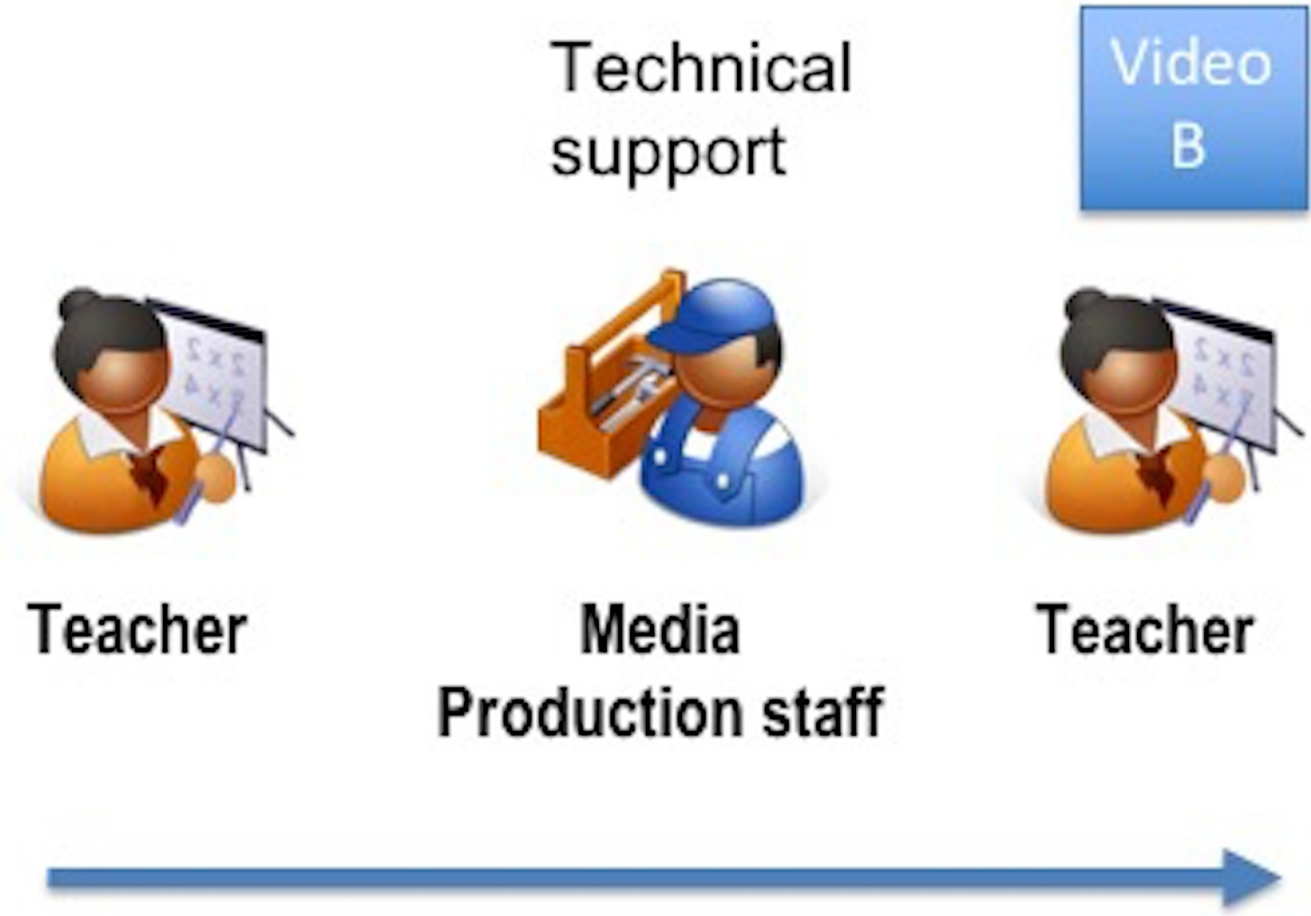
Process to produce video B (not accessible).

The process to develop the videos was as follows: First, a teacher with a long experience in the creation of learning multimedia materials, but with no specific experience in accessibility, authored a Mathematics video addressing the concept of prime numbers with the support of the staff from the media production unit (video B, not accessible), see Fig 1.

More specifically, the teacher designs the material, writes the script, and produces a set of slides with the static visual information. Then, the teacher records the video with the support from the multimedia production unit, who provide the required technology and assist the teacher during the recording. The recorded multimedia material consists of:

- Video information: the slides (static information) plus the images/text that the teacher manually draws/writes on them and that are recorded by the system (dynamic information). The face of the teacher is also recorded.
- Audio information: the teacher’s voice is recorded as the narration of the video.

**Fig 2:**
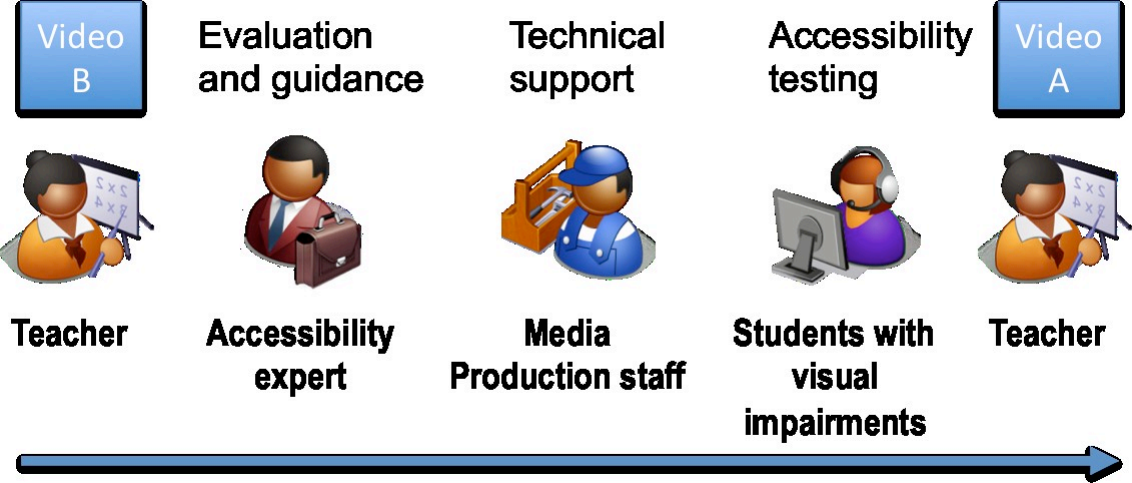
Process to produce video A (accessible).

Then, in order to produce the accessible version of the video (video A), a group of experts in teaching mathematics to visually impaired and blind students identified the accessibility issues of video B, and provided a set of recommendations to make a new video with the same pedagogic objectives, and at the same time accessible for that target group. Then, the teacher designed and recorded a new video in which those recommendations were followed, again with the support of the media production unit. The accessibility specialists assessed the accessibility of video A, and considered that it is accessible for visually impaired and blind students. The overall process is illustrated in Fig 2.

#### Pedagogical features

Both videos A and B have the same learning content and objectives. Through them, the students are taught about the three concepts defined in the 6^th^ grade Mathematics syllabus for the “prime numbers” topic, namely:

- The definition of prime numbers and composite numbers,
- The geometrical interpretation of prime and composite numbers,
- The use of the Eratosthenes sieve to identify the prime and composite numbers.

The video material is to be used with students who have just been through the concept of divisors.

Regarding the Design Principles [45] the production of both videos has been guided mostly by the principles of the CTML [6]. Additionally, we used other features proposed by [8], i.e. synchronizing mathematical handwritten text with teacher’s oral narration, and [12], i.e., “the content was segmented into clear steps; the video contents were connected to previous mathematical knowledge; clear visuals were used when necessary to illustrate key aspects of problems; important elements in problems were highlighted in order to focus student attention; a conversational, relaxed voice was used to engage users; and the length of each clip was kept to a minimum to address issues of limited attention span”. Also, the face of the teacher is displayed on the video, in agreement with [48].

#### Accessibility features

Video B was produced first. Then, its appropriateness to use with students with visual impairments (either low vision or blindness) was assessed by a team of accessibility experts. The reviewers -two math teachers, a computer teacher and a teacher from a non-scientific area- from the Spanish National Organisation for the Blind (Organización Nacional de Ciegos Españoles, ONCE) are experts with experience of at least 25 years in teaching Science and Mathematics to students with visual impairments. Within the review, that included tests with students with different visual conditions, the experts followed the ONCE guidelines for ensuring that digital learning materials are accessible for students with visual impairments [49].

After the review of Video B, the experts proposed the following recommendations in order to improve its accessibility:

- Improve the contrast by changing the color of the pen used by the teacher to write on the screen.
- Use thicker lines for both characters and drawings.
- Increase the size of the pictures in the drawings.
- Verbally explain the visual contents in the video, such as the Eratosthenes sieve and the list of prime numbers.

With the support of the media production unit, the experts’ recommendations were adopted, and a new video called Video A was developed.

First, with the aim of procuring an aural description of the visual information, the teacher wrote a new script for Video A. The only difference with the script of Video B was that the new script included the aural description of visual information that is relevant from the pedagogical viewpoint (see Table 1). As a consequence of the oral description of visual information in video A, its duration is 10 minutes 15 seconds, while Video B’s duration is 8 minutes 43 seconds. During the recording of both videos, the teacher used a tele-prompter to adhere to the script.

**Table 1:** Examples of the differences between Videos A and B, in terms of oral narration and compliance with WCAG 2.0 guideline 1.2.3.

	Definition and examples of prime numbers	Geometrical application of prime/composite numbers	The sieve of Eratosthenes
**Video B**	No list is read out after classifying the first 15 natural numbers into prime or composite numbers.	“Imagine we have 6 square tiles, and think of making a room with those 6 tiles. We can make it like a corridor. And we can also make it like a rectangle”.	Not every crossed out number is read by the narrator (e.g. “[…] and then, we cross out the rest of the numbers”).
**Video A**	After classifying the first 15 natural numbers into prime or composite numbers, the full list of prime numbers smaller or equal to 15 is read out.	“Imagine we have 6 square tiles, one next to the other in line: 1, 2, 3, 4, 5 and 6. And think of making a room with those 6 tiles. We can make it like a corridor. And we can also make it like a rectangle”.	Narrator reads each number that is crossed.

**Fig 3:**
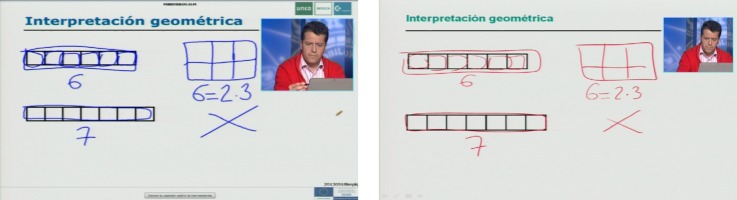
Screenshots of video A (left) and B (right). The person in the image is Emilio Letón, one of the work co-authors, who has given written informed consent for its publication.

Second, in order to optimize the perception of visual information, video A was produced with improved contrast and size of its visual elements (drawings, text and teacher’s image). For doing so, the media production unit modified the visual settings of the recording tool accordingly. Furthermore, both videos (A and B) comply with WCAG guidelines 1.4.1 (Use of Color) and 1.4.8 (Visual Presentation). See Fig 3 and Table 2 for details and samples of the differences between videos A and B.

**Table 2:** Differences of Videos A and B in terms of visual perception.

	Contrast of objects (WCAG 2.0 Guideline 1.4.3)	Size of computer text [50]	Size of handwritten text [50]	Size of the teacher’s image (WCAG 2.0 Guideline 1.4)
**Video B**	Foreground: #FF0000 (the color red) Background: #EBF0E8 (light grayish green) Contrast Ratio of 3.46:1, fails AA level for normal text	Arial 24	Mean height: 22 pixels Thickness: 3 pixels	157 x 177 pixels
**Video A**	Foreground: #2323FF (mainly the color blue) Background: #EBF0E8 (light grayish green) Contrast Ratio of 6.59:1, passes AA level for normal text	Verdana 40	Mean height: 46 pixels Thickness: 4 pixels	209 x 234 pixels

At the end of the process, video A was reviewed by the team of accessibility experts, including tests with blind students and students with low vision, who considered that video A is accessible for students with visual impairments.

## Methodology

### Participants

The participants in this trial were 6^th^ grade students (12-year-ods) attending three schools that belong to the same school system and follow the same curriculum. All participants were non-disabled.

The trial was carried out with the permission of Fuhem (http://www.fuhem.es/), the institution to which the three participating schools belong. The methods and materials were previously agreed on with the teachers, and the director of studies authorized the experience to be included in their program of study. Furthermore, the subject of the exploratory lesson, “prime numbers”, and the time when the research experience took place were agreed on with the teachers of the three schools, according to their program of study for mathematics.

Because participants were minors, the participant consent was handled as follows: each school sent a letter to the students’ parents or guardians, where they were informed about the purpose of the experience, its characteristics, the date, and the researchers’ contact information. Furthermore, the letter informed them that their participation was voluntary, that only anonymous information was to be gathered, and that they had the right to refuse their children’s participation.

Immediately after the experience was carried out, each teacher resumed her/his program of study as usual, including the “prime numbers” lesson.

### Design

The trial was designed to assess the quality of the learning achieved with two types of videos: A and B. Both videos have the same pedagogic content, one of them produced without accessibility in mind (video B), while the other one is accessible for students with visual impairments (video A).

In order to avoid bias, groups were formed from random samples at each school, and were stratified in order to ensure a homogeneous distribution by classroom and by gender in each group. For that purpose, a pack of playing cards that had been previously prepared according to the number of female and male students in each classroom was dealt separately to girls and boys. For girls, we prepared the same number of black suit cards as red suit ones. We did the same for boys. Thus, students were initially divided into two randomly assigned groups, those who got a red suit were to sit in a classroom where Video A would be shown and those with a black suit would sit in a classroom showing Video B.

Both videos A and B were projected simultaneously in the different classrooms. This was the first time the students watched the videos. The visual conditions of the projection [51] were similar in all classrooms: they were made through smart boards (all of them were of the same model), the blinds were closed and the lights were on. Regarding the auditory conditions of the projection (WCAG 2.0 guidelines 1.4.2 and 1.4.7), in each classroom the teacher used the regular settings.

A brief introduction to the experiment was made by each class teacher and one member of the research team. Once the video finished, students were asked to take two tests (objective and subjective) in a maximum of 25 minutes. Both tests are described further in the next section. Finally, the completed questionnaires were collected, and students were thanked for their participation in the experiment.

### Assessment tools

According to [43], asking for the minimum of information required for the purpose is a general principle for designing surveys that is specifically important when participants are children. This, together with the need of running the whole experience within the 45 minutes’ duration of a regular class, were the reasons to keep the questionnaire as short as possible.

As for the objective indicators, we have followed [6,44] that consider retention and transfer aspects of the learning process. The objective test included 10 questions (Q1-Q10), which were agreed on with the teachers. Each question was assigned a maximum grade of 1 point. The aim of this test was to measure the retention and transfer aspects of learning, taking prior knowledge into account:

- Prior knowledge: we are not aiming to assess either prior knowledge in 6^th^ grade Mathematics or in the “prime number” topic, but the students’ knowledge about the atomic concept of “divisor”. It was agreed that this concept would be previously taught by the teacher in a regular class, as it necessarily preceded the concept of “prime number”. It is important to note that both videos include statements that apply the concept of divisor in the very beginning (e.g. “The divisors of 6 are: 1, 2, 3, and 6”, 00 m 45 s). Hence, the purpose of Q1 was to measure prior knowledge about the “divisor” concept.
- Retention: Q2-Q7 questions address the pedagogical contents that are explicitly mentioned in the video, and that are distributed homogeneously throughout the video timeline. Regarding their content validity, these questions cover the three concepts comprised within the “prime numbers” topic, described in the Materials section (pedagogical features), namely: 1) definition of prime and composite numbers, 2) geometrical interpretation of the prime numbers, and 3) the procedure to distinguish prime from composite numbers. These concepts cover the whole “prime numbers” chapter of the 6^th^ grade Mathematics syllabus, and were agreed on with the teachers.
- Transfer: each of the transfer questions (Q8-Q10) covers one different basic concept of the syllabus, hence we assume their content validity.

The subjective test included questions on the video’s image and sound quality, comprehensibility, duration, and helpfulness for learning, as well as overall satisfaction with the video. Regarding its content validity, the questions address each of the principles of Universal Design that are applicable to our research, namely:

- Perceptible information: Question 1 (“Clearly seen”) addresses the perception of visual information, and question 2 (“Clearly heard”) deals with the perception of auditory information.
- Low physical effort: Question 3 (“Easy to understand”) focuses on the cognitive effort, and question 4 (“Short”) focuses on using “the design efficiently, comfortably and with a minimum of fatigue”.
- Equitable use: Question 5 (“Helpful”) addresses the usefulness aspect, while question 6 (“I liked it”) addresses the appealing aspect of the principle.

Its design and piloting followed the recommendations in [43] for measuring children’s opinions of technology. In line with this work, a Visual Analogue Scale based around a 1–5 Likert scale was used. Children were asked to indicate the extent to which they agreed with the corresponding statements by circling one of 5 “smileys” ranging from strongly agree (5), agree (4), normal (3), disagree (2), strongly disagree (1). [43] considers that this method is easy to complete, quick to complete, requires limited reading ability, and requires no writing. In order to reduce the satisficing effect, the test has been designed and piloted so that the questions are especially easy to understand, and the answers are easy to complete.

Furthermore, in agreement with [43], the objective and subjective questions were piloted with 20 students, in the same conditions of the large-scale trial. The aim of this pilot experiment was to identify if there were test questions that were not properly understood or had inappropriate language for the age of the students. As a result of this pilot experiment, abstract questions were replaced by specific questions (for example, "Given any number, how many divisors does it have at least?" was replaced by “What are the divisors of the number 17?”) and negative questions were replaced by affirmative questions (for example, "can you give an example of an odd number that is not prime?" for "can you give an example of an odd number that is composite?). Also the term “product” was replaced by “multiplication”. Another objective of the pilot experiment was to check that the time needed to complete the survey was appropriate.

The objective and the subjective tests are included in S1 Student Test.

## Results

A total of 228 participants (112 males and 116 females) took part in the research experience. 117 participants watched video A (accessible) and 111 participants watched video B. The data have been analyzed with the R software.

The data measured is presented using the mean and the median as centralized statistics, and the standard deviation (SD) and the interquartile range (IR) as dispersion statistics. To compare the differences in quantitative variables between the groups, we established to use a parametric test (Student’s t-test) if the assumptions of normality and homoscedasticity were met, and a nonparametric test (Mann-Whitney test) if any of these assumptions were not met. To assess the normality we used the Shapiro-Wilk statistic, and for the homoscedasticity we used the Levene test. To compare differences in qualitative variables between the groups we used the Chi-square test. In all the tests the significance level of 0.05 was taken. The reliability of the assessment tools, in terms of internal consistency of their questions, was measured through Cronbach’s alpha.

The time it took participants to take the tests as described in Table 3 confirms that the time allowed within the experiment was appropriate.

**Table 3:** Time (m) to complete the test by group.

Group	Time (Mean)	Time (Median)	Time (SD)	Time (IR)
A	14.29	14.00	4.21	6.00
B	14.38	14.00	4.20	5.00

### Results of the objective test

#### Results for retention and transfer

**Fig 4:**
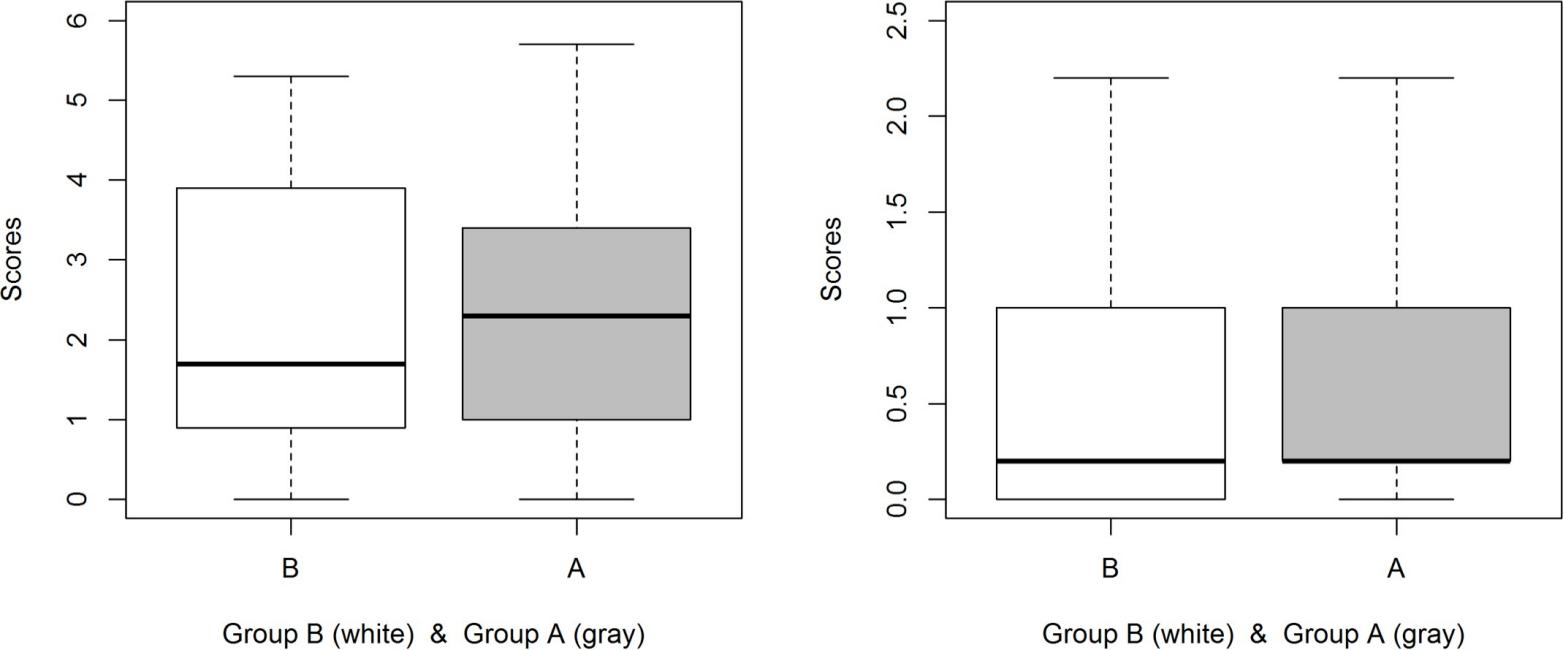
Scores obtained in the retention (left) and transfer questions (right) by group.

Regarding the reliability, Cronbach’s alpha is 0.761 for the quantitative questions (Q2-Q10), 0.747 for the retention questions (Q2-Q6), and 0.322 for the transfer questions (Q7-Q10). The scores obtained by participants in the retention questions are presented in Table 4. There is no statistical significance in the scores of the retention questions with respect to the two groups (Mann-Whitney, p = 0.875), although the median in group A (2.30) is higher than the median in group B (1.70), see Fig 4.

**Table 4:** Scores obtained in the retention questions (SRQ) by group.

Group	SRQ (Mean)	SRQ (Median)	SRQ (SD)	SRQ (IR)
A	2.30	2.30	1.46	2.50
B	2.28	1.70	1.55	3.00

The scores obtained by participants in the transfer questions are presented in Table 5. There is no statistical significance in the scores of the transfer questions with respect to the two groups (Mann-Whitney, p = 0.339), being the median in group A (0.200) equal to the median in group B, see Fig 4.

**Table 5:** Scores obtained in the transfer questions (STQ) by group.

Group	STQ (Mean)	STQ (Median)	STQ (SD)	STQ (IR)
A	0.58	0.20	0.60	0.80
B	0.50	0.20	0.56	1.00

#### Results for retention and transfer, when prior knowledge is considered

Given that question Q1 measures the prior knowledge about the “divisor” concept, which is key for understanding both videos, we have analyzed the scores by group (A and B), and by the results obtained in Q1 (see Table 6). We only considered two groups according to the score obtained in the previous knowledge question: those students who scored 0, and those with a score > 0.

**Table 6:** Scores obtained in the retention questions (SRQ), by group and by score obtained in the previous knowledge question (SPKQ): SPKQ = 0 (PKQ0), SPKQ>0 (PKQ1).

Group	SRQ in PKQ0 (Mean)	SRQ in PKQ1 (Mean)	SRQ in PKQ0 (Median)	SRQ in PKQ1 (Median)	SRQ in PKQ0 (SD)	SRQ in PKQ1 (SD)	SRQ in PKQ0 (IR)	SRQ in PKQ1 (IR)
A	1.29	2.74	0.95	2.80	1.02	1.42	1.18	2.20
B	1.43	2.69	1.05	2.50	0.94	1.62	1.00	3.00

**Fig 5:**
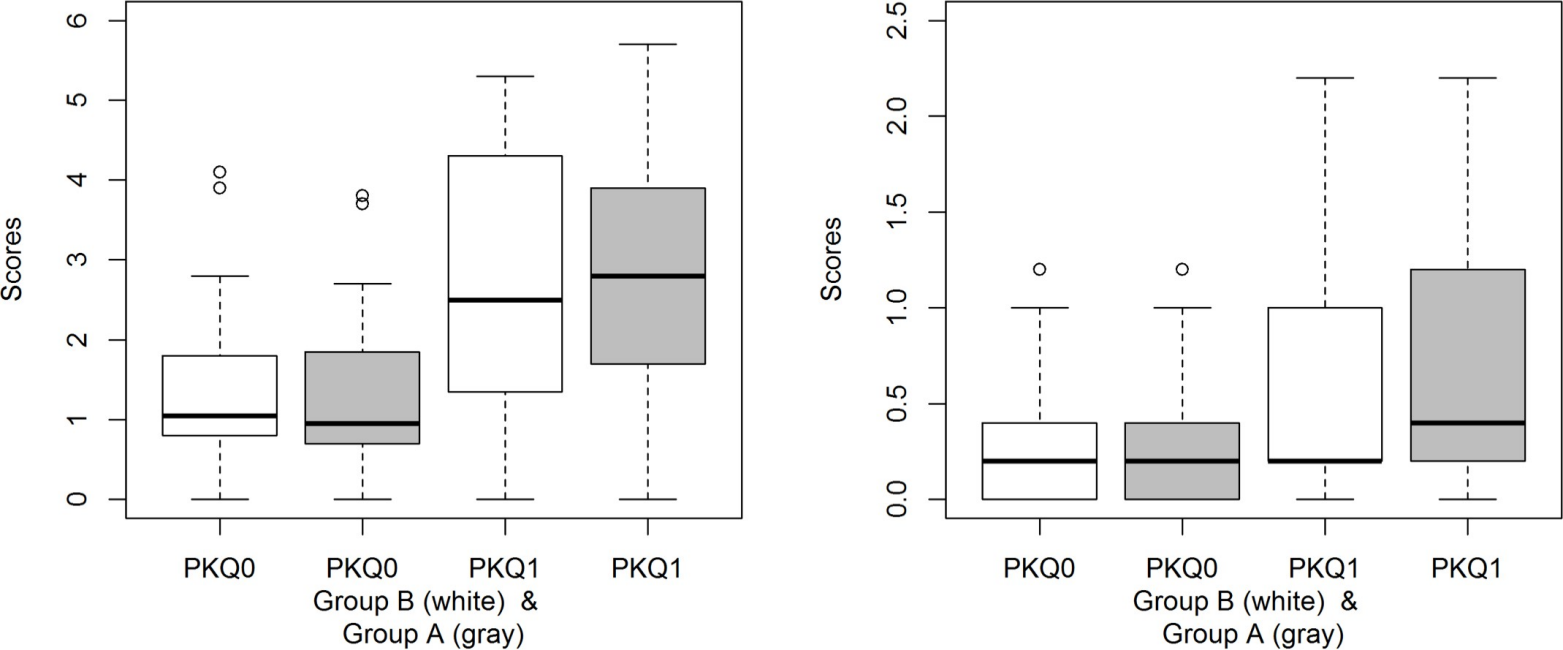
Scores obtained in the retention (left) and transfer questions (right), by group and by score obtained in the previous knowledge question (PKQ): PKQ = 0 (PKQ0), PKQ>0 (PKQ1).

As expected, students who had a better previous knowledge scored higher in the retention test. Among them, the median in group A (2.80) was higher than the median in group B (2.50), see Fig 5, although these results were not statistically significant (Mann-Whitney, p = 0.953). In the case of individuals without previous knowledge the results were again not significant (Mann-Whitney p = 0.412) with a median in group A of 0.95 and 1.05 for group B, see Fig 5.

We analyzed the scores obtained by participants in the transfer questions, both by group (A and B) and by the score obtained in the previous knowledge question (see Table 7). In both cases (PKQ = 0 and PKQ>0) the results were not statistically significant (Mann-Whitney, p = 0.570 and p = 0.403, respectively).

**Table 7:** Scores obtained in the transfer questions (STQ), by group and by score obtained in the previous knowledge question (SPKQ): SPKQ = 0 (PKQ0), SPKQ>0 (PKQ1).

Group	STQ in PKQ0 (Mean)	STQ in PKQ1 (Mean)	STQ in PKQ0 (Median)	STQ in PKQ1 (Median)		STQ in PKQ0 (SD)	STQ in PKQ1 (SD)	STQ in PKQ0 (IR)	STQ in PKQ1 (IR)
A	0.33	0.69	0.20	0.40		0.36	0.65	0.40	1.00
B	0.31	0.59	0.20	0.20		0.40	0.61	0.40	0.80

#### Results of the subjective test

Regarding the reliability of the subjective questions, their Cronbach’s alpha is 0.807. Tables 8 and 9 present the accumulated percentages, and the mean ratings to the statements for students who chose either “strongly agree”, “agree” or “normal”, respectively. In the case of how participants rated the videos against the statement “I liked the video” we found statistical difference between the groups A and B (Chi-squared, p = 0.042). For that question, the accumulated percentages for students who chose either “strongly agree”, “agree” or “normal” was 87.9 (group A) and 78.9 (group B), see Table 8. Furthermore, in the case of the statements “The video could be seen clearly”, “The video could be clearly heard”, “The video was easy to understand”, “The video has been short”, and “The video has helped me learn” there was no statistical difference between groups A and B (Chi-squared, with p = 0.116, p = 0.461, p = 0.255, p = 0.214, p = 0.710, respectively).

**Table 8:** Accumulated percentage (%) for students who chose either “strongly agree”, “agree” or “normal”, by group.

Group	Clearly seen	Clearly heard	Easy to understand	Short	Helpful	I liked it
A	94.8	95.7	74.6	83.5	85.3	87.9
B	97.2	91.6	73.1	90.7	87.2	78.9

**Table 9:** Mean ratings (1–5) in the subjective questions by group.

Group	Clearly seen	Clearly heard	Easy to understand	Short	Helpful	I liked it
A	4.03	4.22	3.31	3.47	3.99	3.66
B	4.16	4.17	3.24	3.62	3.95	3.60

## Discussion

To assess the learning experience with each of these two multimedia materials, an anonymous evaluation was conducted through two sets of questions, aiming at assessing the quantitative and qualitative indicators, correspondingly. The internal consistency of the questions has been measured through Cronbach’s alpha, with acceptable values (>0.7) except for the transfer questions (0.322). With respect to the content validity, the quantitative questions cover all the content in the “prime numbers” chapter of the 6^th^ grade Mathematics syllabus, and were agreed on with the teachers. The qualitative questions address each concept within the Universal Design principles that are applicable to our study.

The learning experience was carried out in real world settings, i.e., real classrooms where smart boards were used to provide image and sound to the students, following [4] we used a random assignment to the groups in order to fill the gap of previous studies noticed in [12].

Regarding the “Perceptibility of information”, the accessibility experts consider that only video A is fully perceptible for people with visual impairments. No differences in the material perception by the students (i.e., subjective test questions “The video could be seen clearly”, “The video could be clearly heard”) came out during the experience between videos A and B, which is not surprising as all who took part in the experience were non-disabled.

As for the “Equality of use”, there are no statistical differences either in the retention or in the transfer questions of the two groups of students. However, the score median in the retention questions is higher in the case of the group using the adapted video (2.30 vs. 1.70, see Table 4). In the case of students with better previous knowledge (i.e., knowledge about the “divisor” concept), they obtained better results both in the retention and in the transfer questions. Furthermore, within the group of students with better previous knowledge, those who watched the adapted video scored better both in retention (median of 2.80 vs. median of 2.50, see Table 6) and in transfer (median of 0.40 vs. median of 0.20, see Table 7), although these results are not statistically significant. In line with our results, [33] describe an eye-tracking study whose results confirm that audio description focuses the attention of non-disabled children, especially when new concepts are introduced, just as in our case.

Furthermore, the “Equality of use” also implies that design should be appealing to be used for all. In this respect, the analysis of the students’ ratings of the statement “I liked the video” in the subjective test indicates that the adapted video was found significantly more attractive than the original counterpart (p = 0.042), see Table 8. Our results are in line with the conclusions of the literature review in [42] indicating that higher levels of saturation and lightness of the color create a positive attitude toward the material, and that the color red should be avoided, see Fig 3 and Table 2. Our results also confirm those by [39], who concluded that audio description did not detract from the enjoyment of a TV program for elderly users with normal vision.

Regarding the “Low physical effort” principle, there are two effects derived from implementing the aural description of visual information in the adapted video (A), which was required by the accessibility experts to ensure the material’s perceptibility. On the one hand, the adapted video is necessarily longer (10 minutes 15 seconds vs. 8 minutes 43 seconds), while experts’ opinion says that shorter videos maximize learners’ concentration [47]. On the other hand, when producing the aural description we have adopted the strategy of writing a new script and recording a new video, rather than including the audio-description in the pauses of the dialogue, see Table 1. By doing so, we ensured that the description was concurrent with the visual information and therefore minimizing the cognitive load, accordingly with [44] and with [40]. In any case, and as stated above, using the adapted video did not hinder the learning process in our experience.

## Limitations of the study

We acknowledge that the lack of internal consistency of our transfer questions limits the validity of the results obtained for the variable “transfer”. Hence, these questions should be redesigned in order to improve Cronbach’s alpha. Furthermore, considering that this was the very first time that the participants faced the concept of prime numbers, we could expect that most of the learning gained would be in the retention side, and that it was too early to measure the learning transfer. An alternative approach for measuring differences in learning transfer may require the re-design of the learning experience, with additional learning activities are included after the students use the video, and before they take the transfer quiz [48].

Other limitations derived from the scope of our study have to do with the type of multimedia content we have considered, its application in different curricula, methods of inclusion, levels of students’ achievement, etc.

## Conclusions and future work

In this paper we apply and discuss a subset of the Universal Design criteria (specifically those addressing the needs of students with visual impairments) in order to produce and use Mathematics multimedia for primary education students. Also, this paper has analyzed how applying accessibility criteria for people with visual disabilities when creating Mathematics multimedia material, can affect the amount and depth of learning for non-disabled students, as well as the organizational implications of adopting Universal Design. To our knowledge it has never been studied before.

Our study is in agreement with others that indicate that inclusive education shows neutral to positive effects on students both with and without disabilities, e.g. generic studies such as [31,32], as well as studies on learning through multimedia, such as [33]. In particular we have found statistical significance in the preference of accessible multimedia. So, we can say that our study provides scientific evidence towards demonstrating that everybody can benefit from the recommendations for developing good quality, accessible multimedia material. Such evidence is one of the critical components of a successful collaboration between teachers and specialist educators (accessibility experts in our case) [29].

Furthermore, this should encourage the increase of awareness about accessibility as well as the creation and adoption of guidelines that suit the emerging ways to produce multimedia in primary education (e.g., [9]). Having such guidelines seems to be especially necessary in this context, as most primary education teachers who produce their own videos can’t rely on accessibility experts. Adopting those guidelines from the very beginning of the authoring process complies with applicable best practices in multimedia learning [44] and universal design of multimedia learning materials [40]. These measures need the support from policy-makers since, in agreement with [28], “policy can go far in articulating an organizational commitment on accessibility, and building an ‘accessibility-first’ mindset across the organization”. Authors of [30] state that teachers should have the skills necessary to use effective practices (“those validated through rigorous research”) and school managers should have tools to support both teachers and students. As a prerequisite for the latter, college and university preparation programs that train teaching professionals should be improved [52].

Regarding the measurement of the learning transfer, the set of questions should be modified in order to increase its internal consistency. We believe that the number of questions should be increased, and that more specific questions should replace the current ones, which might result a bit abstract for primary education students. Furthermore, the use of additional learning activities could contribute to a better training for the students before they face the transfer quiz, and to measure potential differences among the two groups. Additionally, we need to note that the videos used in our study do not include either complex visual information or complex mathematical expressions. Accommodating any of these contents to visually impaired students might require other techniques that are not considered in our work. Hence, further research is needed for understanding the implications of Universal Design in the case of videos with those types of contents. Still, more research is needed to answer that question bearing in mind different curricula, methods of inclusion, levels of students’ achievement, etc. As an improvement to our research experience, we are planning to use affective computing to complement the information provided by subjective tests, as well as to explore the use of interactive learning objects [53], including interaction with annotated videos, see [54] and [55].

## Supporting information

S1 Student test Appendix. This file contains the test used during the learning experience.
(DOCX)

S1 Data Data file. This file contains the data collected during the learning experience.
(XLS)
