# Supplementary material for: Accessible mathematics videos for non-disabled students in primary education
Source: PLoS One. 2018 Nov 28;13(11):e0208117. doi: 10.1371/journal.pone.0208117 (PMC6261620; doi:10.1371/journal.pone.0208117)
Supplement: S1 Student test — This file contains the test used during the learning experience. (DOCX) [file pone.0208117.s001.docx]

# Appendix. Student test

## Objective test

Q1. What are the divisors of the number 17?

Q2. Define in your own words what is meant by a prime number.

Q3. How many numbers are both even and prime? Explain your answer.

Q4. Can you give an example of an odd number that is composite? Explain your answer.

Q5. Imagine you have 13 square tiles. Draw the floor of a room using the 13 tiles. Explain your answer.

Q6. What is the name of the procedure to give a list of prime numbers?

Q7. Give a list of the first 10 prime numbers. Explain your answer.

Q8. If you take two prime numbers and add them, will the result always be a prime number? Explain your answer.

Q9. If you take two prime numbers and multiply them, will the result always be a prime number? Explain your answer.

Q10. Can you write 30 as a multiplication of three prime numbers? Explain your answer.

## Subjective test

Now we would like to know what you think about the video you just saw. Please circle the option you prefer for each sentence:

| 1. The video could be seen clearly | \| 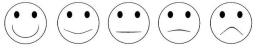  Strongly agree \| 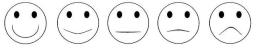  Agree \| 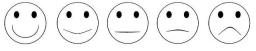  Normal \| 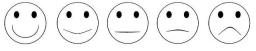  Disagree \| 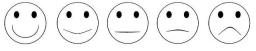  Strongly disagree \| \| --- \| --- \| --- \| --- \| --- \| |
| --- | --- | --- | --- | --- | --- | --- |
| 1. The video could be clearly heard | \| 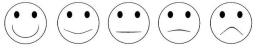  Strongly agree \| 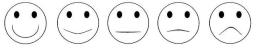  Agree \| 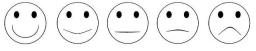  Normal \| 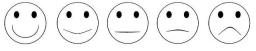  Disagree \| 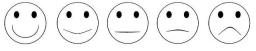  Strongly disagree \| \| --- \| --- \| --- \| --- \| --- \| |
| 1. The video was easy to understand | \| 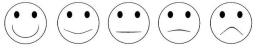  Strongly agree \| 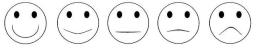  Agree \| 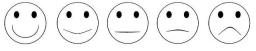  Normal \| 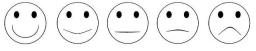  Disagree \| 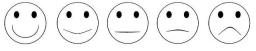  Strongly disagree \| \| --- \| --- \| --- \| --- \| --- \| |
| 1. The video was short | \| 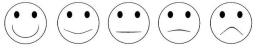  Strongly agree \| 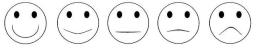  Agree \| 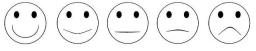  Normal \| 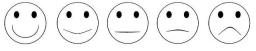  Disagree \| 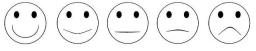  Strongly disagree \| \| --- \| --- \| --- \| --- \| --- \| |
| 1. The video helped me learn | \| 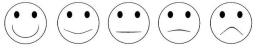  Strongly agree \| 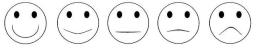  Agree \| 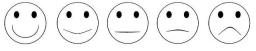  Normal \| 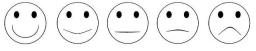  Disagree \| 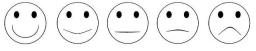  Strongly disagree \| \| --- \| --- \| --- \| --- \| --- \| |
| 1. I liked the video | \| 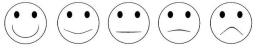  Strongly agree \| 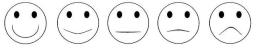  Agree \| 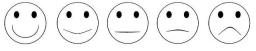  Normal \| 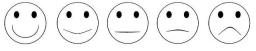  Disagree \| 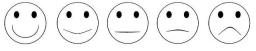  Strongly disagree \| \| --- \| --- \| --- \| --- \| --- \| |
